# CXCR5^+^CD8 T cells: Potential immunotherapy targets or drivers of immune-mediated adverse events?

**DOI:** 10.3389/fmed.2022.1034764

**Published:** 2022-10-13

**Authors:** Christi N. Turner, Genevieve N. Mullins, Katrina K. Hoyer

**Affiliations:** ^1^Quantitative and Systems Biology Graduate Program, University of California, Merced, Merced, CA, United States; ^2^Department of Molecular and Cell Biology, School of Natural Sciences, University of California, Merced, Merced, CA, United States; ^3^Health Sciences Research Institute, University of California, Merced, Merced, CA, United States

**Keywords:** immunotherapy (ICB), immune-related adverse events (IRAE), immune-mediated adverse events (IMAE), CD4 T follicular helper (CD4 Tfh), T follicular helper (Tfh), CD8 Tfc, CXCR5^+^ CD8 T cell, autoimmune disease

## Abstract

CXCR5^+^CD8 T cells have attracted significant interest within multiple areas of immunology, cancer, and infection. This is in part due to their apparent dual functionality. These cells perform as cytotoxic cells in a variety of infection states including LCMV, HBV, HIV and SIV. However, CXCR5^+^CD8 T cells also associate with B cells in peripheral organs and function to stimulate B cell proliferation, antibody/B cell receptor class-switch, and antibody production. CXCR5^+^CD8 T cells are similar to CXCR5^+^CD4 T follicular helpers in their genetic make-up, B cell interactions, and functionality despite possessing elevated programmed cell death 1 and cytotoxic proteins. Within cancer CXCR5^+^CD8 T cells have risen as potential prognostic markers for overall survival and are functionally cytotoxic within tumor microenvironments. In inflammatory disease and autoimmunity, CXCR5^+^CD8 T cells are implicated in disease progression. During viral infection and cancer, CXCR5 expression on CD8 T cells generally is indicative of progenitor memory stem-like exhausted cells, which are more responsive to immune checkpoint blockade therapy. The use of immune checkpoint inhibitors to overcome immune exhaustion in cancer, and subsequent consequence of immune adverse events, highlights the dual nature of the cellular immune response. This review will detail the functionality of CXCR5^+^CD8 T cells in cancer and autoimmunity with potential repercussions during immune checkpoint blockade therapy discussed.

## Introduction

**Figure 1:**
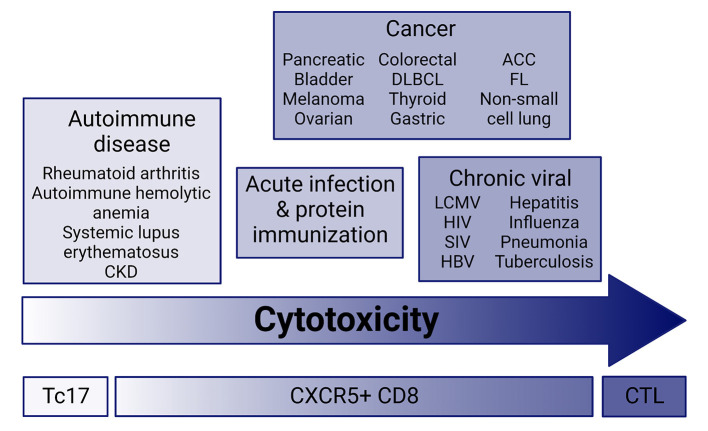
Cytotoxic potential across disease. CXCR5^+^CD8 T cells have a range of cytolytic potential in infection, cancer, and autoimmune disease. Current research suggests that cytolytic and effector molecule expression ranges from very low in autoimmune disease similar to Tc17 effector cells, and high in chronic infection and cancer. CXCR5^+^CD8 T cells from acute infection and immunization with protein maintain an intermediate cytolytic potential. CKD, chronic kidney disease; ACC, Adenoid cystic carcinoma; FL, follicular lymphoma; DLBCL, diffuse large B cell lymphoma; CTL, cytotoxic T lymphocyte; Tc17, IL-17-secreting CD8 T cells.

In the past decade research has focused on elucidating the regulation and functionality of CXCR5^+^ T follicular cells (Tfh) during infection, cancer, and autoimmune disease. CXCR5^+^ T cells interact with germinal center B cells and initiate differentiation into antibody-producing (plasma) cells or memory formation through proliferation, somatic hypermutation, and class-switch recombination (1–6). CXCR5^+^CD4 T follicular helpers (CD4 Tfh) have received most of the limelight for their contribution to germinal center migration, functionality, and B cell help within infection, cancer, and autoimmunity (6–9). CXCR5^+^CD8 T cells have slowly come into focus with their unique ability to provide B cell help within germinal centers similar to CD4 Tfh and also maintain a cytolytic capacity in infection, autoimmunity and tumor microenvironments resembling CD8 T effector cells (Figure 1) (1–5, 10, 11). This mini-review focuses on CXCR5^+^CD8 T cells in cancer, and implications for immune-mediated adverse event (IMAEs) development in patient immunotherapy treatments.

## CXCR5^+^CD8 T cell cytotoxicity and cancer

Tumor microenvironments contain an abundance of innate and adaptive immune cells ranging from tissue-resident cells, such as macrophages, to migratory cells, such as T and B lymphocytes, each with a specific purpose (12). CD8 T cells traditionally activate and differentiate into cytolytic effectors responsible for killing virally infected and cancerous cells. Cytotoxic CD8 T cells are defined by the ability to induce antigen-specific apoptosis of target cells (13). Cytotoxicity is mediated by granzymes, perforin, CD107a, IFNγ, TNFα, TNFβ, and FasL and regulated and defined by key transcription factors, including Blimp-1, eomesodermin (eomes), T-bet, Id1, Id2, and Id3 (14). CXCR5^+^CD8 T cells localize within blood circulation and tumor microenvironments of cancer patients (15–18). T follicular cells produce varying levels of IL-21 and effector molecules within blood circulation compared to lymph nodes and organs, but their ability to assist B cells remains unaltered indicating that T follicular cell functionality may not be site specific (9, 10).

CXCR5^+^CD8 T cells have cytotoxic and proliferative capacity within cancer ranging from liquid to solid tumors. CXCL13 upregulation by tumors induces migration, signaling and functional changes by CXCR5-expressing immune cells, but the specific functional capacity of CXCR5^+^CD8 T cells versus other CXCR5-expressing cells, including B cells and tumors, is poorly characterized in cancer. CXCR5^+^CD8 T cells from human peripheral blood mononuclear cells, tumor tissues and tumor associated lymph nodes upregulate effector molecules such as IFNγ, TNFα, granzyme B, and perforin compared to CXCR5^−^CD8 T cells (10, 17–28). CXCR5^+^CD8 T cells upregulate CD107a, proliferate, and induce specific cell lysis of *in vitro* co-cultured tumor cells (17, 19, 21, 22, 24, 25). CXCR5^+^CD8 T cell infiltration of hepatocellular carcinomas generate a robust anti-tumor response in association with B cell antibody production, through IL-21 production, that correlates with a reduction in early tumor recurrence, and is not associated with peritumoral liver or blood tissues (29). This demonstrates the importance of CXCR5^+^CD8 T cells within the tumor microenvironment and surrounding tissues to patient outcomes and identifies possible tumor eradication mechanisms utilized by CD8 T cells. Furthermore, CXCR5^+^CD8 T cells appear resistant to immune checkpoint blockade therapy (ICB) induced apoptosis compared to susceptible CXCR5^−^CD8 T cells and, instead, demonstrate an effector-like phenotype in infection and chronic lymphocytic leukemia (11, 27). CXCR5^+^CD8 T cells maintain resistance to immune modulation in spite of high program cell death-1 (PD-1) expression, an inhibitory receptor, that is a marker of Tfh. PD-1 on CD4 Tfh retains these cells within germinal centers, with their localization regulated by PD-L1 ligand expression on B cells (6, 30). Since CXCR5^+^CD8 T cells are similar in gene regulation, action, and surface protein expression to CD4 Tfh this mechanism may also regulate CXCR5^+^CD8 T cell homing to germinal centers of lymphoid organs and tertiary lymphoid structures in tumors.

## CXCR5^+^CD8 T cells and markers of exhaustion in infection, cancer, and autoimmunity

T cell exhaustion is defined by successive upregulation of inhibitory receptors on CD8 and CD4 T cells in the presence of chronic antigen stimulation in cancer, infection, and autoimmune disease. T cell exhaustion status is sequential and reversible with a phenotypic range from dysfunctional T effector cells to memory progenitors with cytolytic ability to terminal exhaustion with unknown capacity for functional reactivation (31, 32). Under high antigen and inflammation, a step-wise process of inhibitory receptor activation, effector cytokine reduction, and cytolytic effector downregulation on CD8^+^ T effectors leads to two exhaustion stages, pre-exhausted (Tpex) and terminally exhausted (Tex) (33). Tpex cells are defined by PD-1^int^TCF1^hi^ and Tex cells by PD-1^hi^TIM3^hi^ expression (34). Subsequently, nine Tex subtypes were identified through high-dimensional mass cytometry suggesting that transition between Tpex and Tex involves multiple stages or that multiple inhibitory receptors have overlapping function in driving terminal exhaustion (35). Initiation of exhaustion to Tex begins with loss of IL-2 signaling leading to decreases in TNFα and perforin (32, 36). Cytolytic activity of CD107a degranulation is initially maintained along with regulatory cytokines, such as IL-10, which may upregulate and prompt the exhaustive state. Finally, it is thought that some Tex cells, such as CD8 tumor-infiltrating lymphocytes, undergo activation-induced cell death (AICD) following the loss of proliferative capacity (33). This cell death is FAS ligand activated by FAS upregulation on tumor infiltrating lymphocytes and FAS ligand on adjacent tumor cells (37). AICD associates with Tfh and regulates peripheral tolerance, and AICD loss leads to autoimmune progression possibly due to release of self-reactive lymphocytes from cell death mechanisms (6, 38). In tumor microenvironments, ICB T cell reactivation reduces AICD influence potentially enabling self-reactive Tfh activation and driving IMAEs (39, 40).

**Figure 2:**
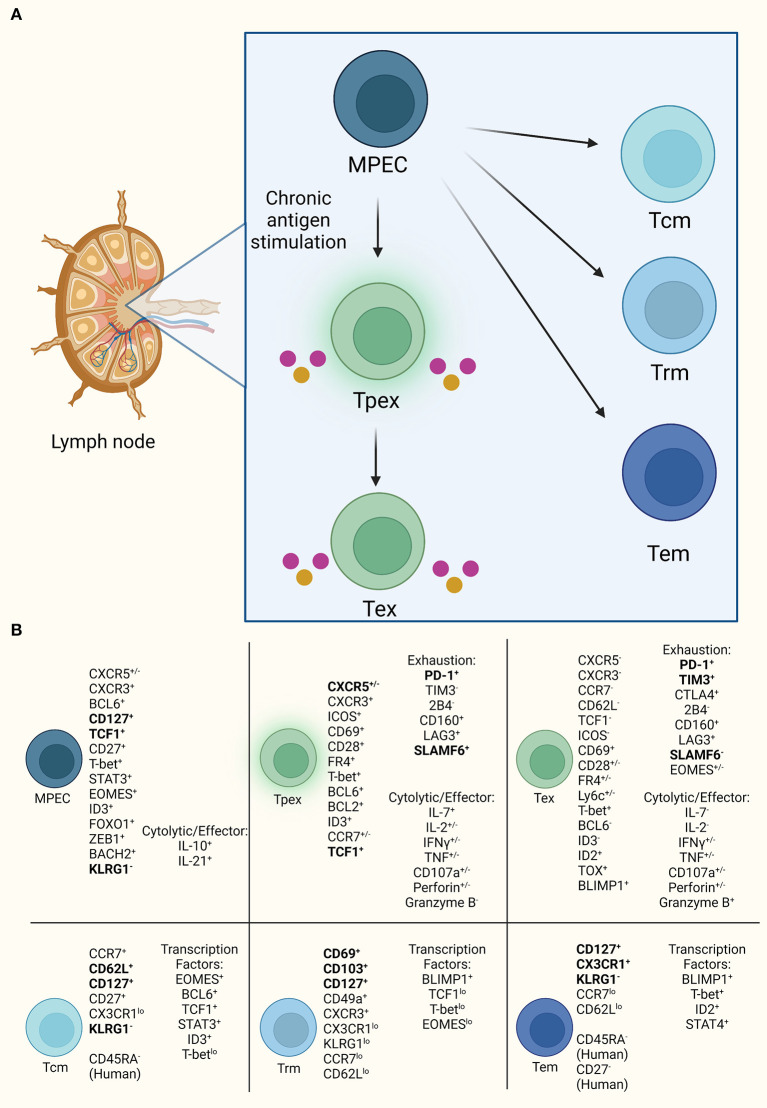
CXCR5^+^CD8 T cell progenitor lineages. **(A)** CXCR5^+^CD8 T cells resemble progenitor cells with self-renewal, such as MPECs, that are capable of diverging into two lineages of either exhausted T cells under high antigen exposure or memory cells with effector functionality. **(B)** Established markers defining CD8 T cells for each subset diverging from MPEC. Bold indicates consistently used markers for population identification. Also, listed are the currently demonstrated cytolytic and effector molecules in exhausted subsets.

PD-1 is the canonical marker of T cell exhaustion and a marker of germinal center immune cells. In infection, PD-1 upregulation induces functional CD8^+^ T cell exhaustion resembling a Tpex cell type (6, 11). CD8^+^PD-1^+^ T cells are heterogeneous across infection, cancer, and autoimmunity based on identification of multiple T cell subsets within each disease (10, 41–43). CXCR5^+^CD8 T cells appear functional with effector/cytolytic capacity in cancer despite high PD-1 expression. CXCR5^+^CD8 T cells are also described as memory precursor effector cells (MPECs) in various settings (Figure 2; Supplementary Table S1) (11, 27, 44–46). MPECs express CD127, TCF1 (TCF7), and T-bet, lack KLRG1, and produce low effector cytokines (33, 47). These cells persist when transferred generating memory and terminally exhausted cells (33, 47). CXCR5^+^CD8 T cell subset identified as TCF1^+^PD-1^+^TIM3^low^ maintain low cytotoxic capability (27, 45), resembling a Tpex subset with limited proliferation but cytokine production (27, 34, 45). Due to their self-renewal capacity and responsiveness to anti-PD-1 therapy these cells have potential as therapeutic targets (11, 46). A tissue resident exhausted progenitor population expressing TCF1 and cytolytic effector molecules is a self-renewing precursor to circulatory and terminally exhausted populations in LCMV and cancer (44). Upregulation of multiple inhibitory receptors regulate the pathway to Tex, including PD-1, TIM3, CTLA4, Lag3, 2B4 in various combinations. Within LCMV infection, CXCR5^+^TCF1^+^TIM3^−^ Tex expand after PD-1 blockade into CXCR5^−^Tcf1^−^Tim3^+^ Tex cells, and in other murine and human settings less exhaustive states exist such as PD-1^lo^KLRG1^hi^TIM3^lo^ and CXCR5^+^PD-1^+^Tim3^−^ that maintain cytolytic capacity (11, 48, 49). CXCR5^+^TCF1^+^TIM3^−^ Tex is possibly a memory population that upon functional reactivation becomes terminally exhausted by upregulating the inhibitory receptor, TIM3, as a secondary means of inhibition in high antigen environments, whereas less exhaustive states with downregulated TIM3 expression demonstrate highly functional CXCR5^+^CD8 T cells.

CXCR5^+^CD8 T cell heterogeneity also exists within cancer and complicates our use of therapies directed at CD8 T effectors (44). CXCR5^+^ T cell subset variation is found across solid and liquid tumors, from non-small cell lung cancer to follicular B cell non-Hodgkin's lymphoma, where PD-1 and TIM3 expression creates diverging populations (10, 50, 51). In hepatocellular carcinoma, follicular lymphoma, thyroid and high-grade serous ovarian cancer, PD-1 is upregulated on CXCR5^+^CD8 T cells while TIM3 and CTLA4 are downregulated compared to CXCR5^−^CD8 T cells possibly indicating a Tpex versus Tex phenotype (15, 21, 24, 25). CXCR5^+^CD8 T cells in pancreatic cancer express both PD-1 and TIM3 suggesting a Tex phenotype that varies from other cancers (17). Inhibitory marker expression also fluctuates by tissue region, for example in hepatocellular carcinoma upregulated PD-1 is expressed in human liver and tumor tissue compared to blood circulation (29). PD-1, TIM3, 2B4, and Lag3 are downregulated in CXCR5^+^CD8 T cells in colon cancer, but within gastric cancer PD-1, Lag3, CTLA4, and Tigit are upregulated (23, 26). Furthermore, differences are associated with human versus murine models of disease, such as in metastatic melanoma where CXCR5^+^PD-1^+^CD8 T cells were identified by single-cell sequencing in humans, but not identified in a B16 melanoma mouse model (10, 52). Additional research in CXCR5^+^CD8 T cell effector status and ability to maintain functionality or diverge into exhaustive states within high antigen environments is needed. Characterization of CXCR5^+^CD8 T cell exhaustion in chronic or high antigen environments is limited for infection, cancer and autoimmunity.

Investigating exhaustion dynamics of CXCR5^+^ T cells within autoimmunity is challenging due to the association of PD-1 as an activating and inhibitory receptor. There is potential for functional differences between CXCR5^+^CD8 and CD4 Tfh cells within germinal centers that may resemble antibody-suppressor CXCR5^+^IFNg^+^CD8 T cells (53, 54). Antibody-suppressor cells, namely CXCR5^+^PD-1^−^CD8 T cells, may activate effector functions on self-reactive B cells and CD4 Tfh cells leading to tolerance and inhibition of plasma cell formation thus halting autoantibody production associated with many autoimmune diseases. Lag3 loss on intra-islet non-obese diabetic CD8 T cells accelerates autoimmune diabetes and highlights Lag3 T cell inhibition differences between autoimmunity, cancer and infection (55, 56). Rao et al. identified a CXCR5^+^PD-1^hi^CD4 population within peripheral blood from rheumatoid arthritis patients, but not within synovial fluid, that expressed Tigit and SLAMF6 suggesting Tfh interaction with B cells, and regulation of inhibitory receptor functionality (57). Within human chronic kidney disease, CCR6^−^CXCR3^−^CXCR5^+^PD-1^+^CD4 and exhausted CD8 T cells are upregulated compared to end-stage kidney disease showcasing exhausted populations in CD4 and CD8 T cells during autoimmunity (58). These studies suggest that non-exhausted and exhausted CXCR5^+^ T cell subsets exist and influence autoimmunity, and their functions may be different than in infection and cancer. ICB induced reactivation of exhausted CXCR5^+^ T cells during autoimmunity may enable new autoimmune disease therapies. Alternatively, since exhaustion downregulates T cell effector function, exhaustion itself may provide an additional inhibitory tool in regulating self-reactive T cells.

## Could CXCR5^+^CD8 T cells initiate the development of immune mediated adverse events?

ICB created a paradigm shift in how we understand cancer, the immune system and treatment modalities for cancer patients. A deeper immune-driven exploration of tumor microenvironments is needed to understand how these treatments enable or disrupt immune cell functions promoting patient survival or tumor progression. ICB reactivates exhausted T cells through downregulating inhibitory receptors and ligands on tumors and immune populations by blocking specific protein receptors such as PD-1, PD-L1, and CTLA4 with monoclonal antibodies (59). By blocking T cell exhaustion, effector T cells regain cytolytic function, including CXCR5^+^CD8 T cells. CXCR5^+^CD8 T cells initiate a proliferative burst of effector T cells following PD-1 immunotherapy administration in LCMV (11). This reinvigoration increases tumor destruction plus prolongs overall survival, approximately 21 months, in many cancer types: melanoma, non-small cell lung, renal cell, head and neck squamous cell, bladder, Merkel cell, hepatocellular, Hodgkin's lymphoma and more (60, 61). Of the patients approved for treatment with ICBs only 15-20% experience cancer remission and majority develop IMAEs or immune-related adverse events following treatment (62).

IMAEs are adverse events resembling organ specific to systemic autoimmunity and cause patients to end treatment early (63). IMAE biomarker development has focused on autoantibodies associated with known autoimmune conditions, such as Anti-Smith, Anti-dsDNA, ACPAs, Rh factor and more, however, these autoimmune biomarkers are poorly defined in cancer and vary in their baseline associations of IMAE outcomes (64). To account for the large autoantibody repertoire for identifying IMAE progression, blood serum from ICB treated patients could be collected pre- and post-treatment to establish a baseline and standardized by high-throughput proteome techniques (65–67). Current studies have utilized microarray, SEREX cDNA library, immunofluorescence, and immunoassay technologies for screening of IMAE associated autoantibodies, such as thyroiditis, hypophysitis, rash, colitis, arthritis, myocarditis, myalgia, and endocrine disorders in response to singular/combination treatments of CTLA4, PD-1 and PD-L1 in melanoma, advanced/metastatic solid tumors, renal cell carcinoma, non-small cell lung, prostate, and bladder cancers (68–73).

CXCR5^+^CD8 T cells correlate with disease-free or overall survival in pancreatic, colon, follicular lymphoma, gastric, high-grade serous ovarian, hepatocellular, and bladder cancers and thus is considered a potential biomarker (15, 17, 20, 23, 25, 26). On the contrary, decreases in disease-free survival in non-small cell lung and salivary adenoid cystic carcinomas are associated with infiltrating CXCR5^+^CD8 T cells (74, 75). Functional studies beyond correlative data and mRNA expression remains lacking as to how these cells benefit or negate a tumor microenvironment. Chemokine, CXCR5, knockdown has proven to have negative prognostic value in certain cancers and cell lines (76). Tumors express CXCR5 and produce its ligand, CXCL13, to recruit B cells and T cells into tumor microenvironments where tertiary lymphoid structures, resembling germinal centers, are developed and antibody production occurs (77–79). CXCR5 promotes proliferation of clear cell renal carcinoma through activating PI3K/AKT/mTOR pathway in the presence of its ligand, CXCL13, and this signaling pathway has also been reported in colon cancer proliferation (76, 80). CXCL13-CXCR5 axis in tumors leads to increased migration, invasion of tumor cells and unfavorable tumor prognostic values in patients, but not in the case of ICB treatment (77, 79, 81). In patients treated with ICB, high CXCL13 expression leads to more favorable tumor outcomes perhaps due to CXCR5^+^CD8 T cells expanding the effector T cell pool or as a consequence of these cells increasing proliferation while resisting apoptosis (11, 82). CXCR5^+^CD8 T cell persistence following ICB may generate a greater antibody repertoire based on their known B cell interactions that could be beneficial for tumor eradication or generate organ specific detriments and IMAEs (1, 79, 83).

CXCR5^+^CD8 T cell ability to initiate IMAEs during ICB remains unknown. These cells are capable of upregulating effector molecules in cancer and lysing tumor cells in co-culture. In autoimmune disease, CXCR5^+^CD8 T cells enable B cell differentiation and exacerbate disease (1, 2). Correlations exist based on immune cell infiltration and IMAE development in patients without a complete understanding of tolerance mechanisms. For example, granzyme B producing CD4 T cells infiltrate thyroids in response to anti-PD1 therapy at a greater frequency than CD8 T cells and are associated with development of mouse and human thyroiditis (84). Production of IL-21, CXCL13, and infiltration by cytolytic CD4 and CD8 T cell infiltrates associate with IMAE development in tumor models and human plasma (85). Whether or not these cells express CXCR5 remains unknown but their ability to produce granzyme B, IL-21, and migrate to CXCL13 warrants further CXCR5^+^ T cell research in IMAE development. A direct link between IMAEs and autoimmune mechanisms is currently unknown. PD-1 elimination and ICB accelerates autoimmunity in murine models of systemic lupus erythematosus and autoimmune diabetes (86, 87). Reversal of inhibitory tolerance mechanisms is capable of reactivating T cell receptors to recognize self-antigen (86). Autoreactive CD8 T cell activation after PD-1 ICB induces autoimmune diabetes in Rip-mOVA mice indicating that loss of CD8 T cell self-tolerance following immune modulation therapies increases autoimmunity (88). Additional studies are needed to determine if self-reactive cells lose tolerance and transform into active effector T cells capable of cytolytic activity on tumor and self-tissues. Also, research is needed to determine if initiation of IMAEs and autoimmunity occur *via* similar or distinct pathways.

## Discussion

CXCR5^+^CD8 T cell research is expanding in multiple areas as they are identified in advanced technologies, such as single-cell sequencing. As with most areas of cancer research heterogeneity between cancer types, biomarkers, and tumor microenvironments creates cumbersome and often contrasting phenotypic conclusions regarding CXCR5^+^CD8 T cell functions. CXCR5^+^CD8 T cells are a newly developing area of study that allow for a unique perspective and growth in the fields of cancer research and immune checkpoint blockade. ICB research is now focused on combining treatment modulations to identify ways to overcome immune-mediated adverse events. Awareness and focus on IMAEs is in its initial stages. How CXCR5^+^CD8 T cells affect the tumor microenvironment, B cell infiltration, autoantibody response, T cell tissue residency, and peripheral organ involvement after ICB remains unknown. Defining functionality in these different areas, particularly identifying how these cells respond in cancer initiation and progression, would provide a major step forward. We speculate that CXCR5^+^CD8 T cells may slowly migrate into circulation after ICB treatment but become primed and activated by dendritic cells in peripheral organs generating an effector phenotype that then infiltrates tissues and establishes IMAEs (45, 86, 89). Because of cancer heterogeneity, identifying CXCR5^+^CD8 T cells requires a robust screen across multiple cancer types to enable a sound and clear prognostic value for using CXCR5^+^CD8 T cells as a biomarker or immune cell therapy target.

Questions about CXCR5^+^CD8 T cell effects on IMAEs remain. CXCR5^+^CD8 T cell ability to interact with B cells and promote antibodies suggests that some CXCR5^+^CD8 T cells, after ICB, could reverse tolerance and initiate autoantibody production. Data from multiple studies now suggests that PD-1 blockade releases self-tolerance constraints increasing autoimmunity. As ICB induces IMAEs, it raises the question of whether IMAEs are caused by autoimmune mechanisms or some other immune dysfunction. With sequencing techniques advancing, teasing out the implications of protein antigens from tumor proteins or self-proteins would delineate if CXCR5^+^CD8 T cells pose a threat to the development of IMAEs (10). Studying CXCR5^+^CD8 T cells using animal models that perpetuate autoimmunity would explain how these cells respond to ICB and if they initiate disease development. An autoimmune disease model, B6*/lpr*, treated with combination ICB in the presence of colon adenocarcinoma develops T cell immune-infiltration of multiple organs with increased tumor growth following steroid treatment (90). Corticosteroids are a drug of choice when patients develop IMAEs but based on this study could result in decreased ICB effectiveness. Promoting tolerance mechanisms in T cells while patients receive ICB may enhance therapy effectiveness. VISTA regulates tolerance mechanisms and its loss induces autoimmune disease in mice, by incorporating a VISTA agonist along with ICB may enhance cancer therapy while eliminating IMAEs (91, 92). In humanized mice, CXCR5^+^CD8 T cells develop after human cord blood hematopoietic stem cell engraftment and HIV infection while maintaining PD-1, cytotoxicity, cytokine production, and homing to peripheral organs (93). These studies provide novel models for elucidating CXCR5^+^CD8 T cells in the context of mouse and human responses, in addition to, existing human tumor resections and peripheral blood mononuclear cells for translational research.

## Author contributions

CT and GM conceptualization, literature evaluation, original draft writing, and generated and visualized figures. KH conceptualization, writing and review, visualization, funding acquisition, and supervision. All authors contributed to the article and approved the submitted version.

## Funding

This work was supported by the National Institutes of Health Grant R15HL146779 to KH.

## Conflict of interest

The authors declare that the research was conducted in the absence of any commercial or financial relationships that could be construed as a potential conflict of interest.

## Publisher's note

All claims expressed in this article are solely those of the authors and do not necessarily represent those of their affiliated organizations, or those of the publisher, the editors and the reviewers. Any product that may be evaluated in this article, or claim that may be made by its manufacturer, is not guaranteed or endorsed by the publisher.
